# Cross-sectional associations between intrinsic and extrinsic motivation for physical activity and body composition in adolescents with obesity

**DOI:** 10.1016/j.obpill.2025.100197

**Published:** 2025-07-19

**Authors:** Fábio de Freitas, Mariana R. Zago, Maria Ângela Antônio, António Videira-Silva, Maria Ângela Bellomo Brandão

**Affiliations:** a Unicamp School of Medical Sciences, Department of Pediatrics, Child and Adolescent Health Graduate Program, Campinas, São Paulo, Brazil; b Unicamp School of Medical Sciences, Department of Pediatrics, Campinas, São Paulo, Brazil; c WHO Collaborating Centre for Adolescent Medicine and Training, Clínica Universitária de Pediatria, Faculdade de Medicina, Universidade de Lisboa, Lisbon, Portugal; d CIDEFES (Research Centre in Sport, Physical Education, Exercise and Health), Universidade Lusófona, Lisbon, Portugal; e CIFI2D (Centre for Research, Training, Innovation, and Intervention in Sport), Universidade do Porto, Porto, Portugal

**Keywords:** Adolescent, Obesity, Exercise therapy, Health promotion, Motivation, Public health

## Abstract

**Background:**

Motivation is essential in turning the intention to engage in physical activity (PA) into consistent action, promoting healthier and more sustainable habits. This study aimed to identify the motivational factors associated with engagement in a PA program and examine the relationship between motivation and body composition in adolescents with obesity.

**Methods:**

Fifty adolescents (60.0 % boys); Median age of 13.6 years (IQR: 2.4); Median BMI z-score of 3.42 (IQR: 1.55) recruited for a PA program were included in this cross-sectional study. All participants completed standardized body composition assessments and an online questionnaire assessing five motivational domains: enjoyment and competence (intrinsic), and appearance, fitness, and social (extrinsic domains).

**Results:**

Fitness emerged as the main reason for engaging in the PA program (6.41 ± 0.81), followed by appearance (5.64 ± 1.43), enjoyment (4.98 ± 1.47), competence (4.55 ± 1.99), and social (3.79 ± 1.85). Enjoyment and competence showed significant negative associations with body weight. (*r*_*p*_ = −0.29, p = 0.045; *r*_*p*_ = −0.32, p = 0.027). Regression analysis identified fitness as a significant predictor for BMI z-score (β = 0.4, p = 0.027).

**Conclusion:**

Extrinsic motives, such as fitness and appearance may drive PA engagement in treatment-seeking adolescents with obesity. However, intrinsic motives, such as enjoyment and competence, may be inversely associated with BMI z-score. Thus, targeting both extrinsic and intrinsic motivation may help to improve adherence and weight outcomes in obesity care. More studies with larger samples are needed to confirm these findings.

## Graphical abstract

**Figure:**
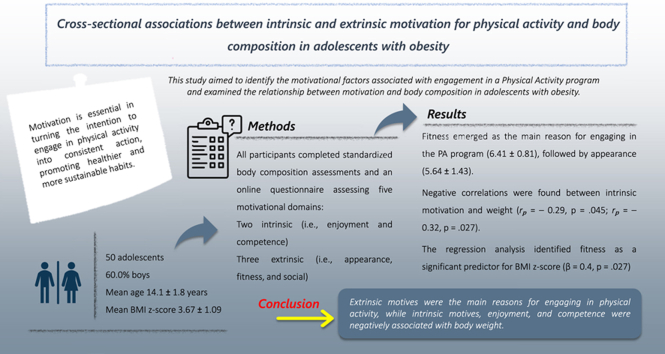

## 1 Introduction

Pediatric obesity is still a global health crisis with significant clinical and public health consequences [1]. Obesity development involves complex biological, psychological, social, and behavioral factors, affecting energy imbalance [2], as well as physical and mental health, in turn, potentially leading to the maintenance of obesity and obesity-related comorbidities in adulthood [3].

Physical activity (PA) may play a relevant role in obesity treatment, not only to energy expenditure, but also due to improved physical, psychological, and social wellness, that are independent of the effect on weight/obesity [4,5]. Despite the well-known benefits, the prevalence of physical inactivity among adolescents remains high [6]. This is particularly relevant since physical inactivity is associated with an increased incidence of obesity and other chronic non-communicable diseases [1,2], and obesity may contribute, in turn, to low levels of PA [7].

Multidisciplinary lifestyle programs for adolescents with obesity have shown positive results [8,9]. However, adolescents with obesity are still a challenge for health professionals, as resistance to adopting healthy behaviors increases with its severity [10].

The transition from late childhood to adolescence is a developmental period marked by well-documented declines in PA [11]. Understanding the specific motivational factors that influence young people to engage in a PA routine can assist in creating more assertive strategies and developing effective interventions for behavior change [12].

Motivation is essential to turning the intention to practice PA into action. It is the engine that drives the adoption of more active habits [12]. The Self-Determination Theory (SDT) suggests that motivation for practicing PA can be intrinsic, where individuals seek pleasure and personal satisfaction from the activity itself, or extrinsic when motivation is influenced by external factors such as social approval or rewards [13]. The Motives for Physical Activity Measure-Revised (MPAM-R) scale, which aligns with SDT, helps us understand the reasons for PA behavior through interrelated factors like enjoyment, the pursuit of competence and challenges, concern for physical appearance, the desire to improve physical fitness, and social motivation [14]. For instance, when individuals engage in PA for intrinsic reasons, such as enjoyment and a sense of competence, they are less likely to quit since they find it interesting, fun, and fulfilling. Conversely, engagement for extrinsic reasons like fitness, appearance, and social status often focuses on gaining rewards or social recognition [15].

Research on the associations between motives for PA practice and engagement in moderate-to-vigorous physical activity (MVPA) among adolescents has highlighted the significance of intrinsic motivation, such as enjoyment and the pursuit of better physical fitness [16]. In other studies, factors like fun and enjoyment emerged as the primary motivations for participating in a mass running event [17]. Adolescents who were more intrinsically motivated, particularly by enjoyment and perceived competence, tended to show increased engagement in PA programs. Nevertheless, extrinsic elements such as social influence and medical guidance may contribute to the observed outcomes [18].

The use of PA/exercise in the context of treatments, particularly for obesity, highlights intrinsic motivation as a determinant factor for behavioral changes related to PA. A lack of interest and enjoyment in the activity can hinder the implementation and maintenance of new habits, potentially resulting in premature termination or insufficient results [12].

This study aimed to identify the motivational factors associated with engagement in a PA program and examine the relationship between motivation and body composition in adolescents with obesity, in order to identify key elements for developing personalized and effective interventions that promote long-term health and well-being.

## 2 Methods

### 2.1 Study design

This quantitative cross-sectional exploratory study used data from participants previously recruited for a non-randomized controlled trial [20].

### 2.2 Participants

A total of 50 adolescents (40 % girls) aged 12–17 years, with obesity BMI z-score ≥2, [19], were included in the present study. Only participants with complete baseline data on the variables of interest (including motivational data) were considered. No further exclusions were applied.

### 2.3 Procedures

Before data collection began, participants and respective guardians were informed about the research objectives and procedures and signed a voluntary informed assent/consent form. After the initial assessments, contact information for the participants was collected so that the questionnaire could be sent. Each participant then received a personalized link via email or text message to access and complete the questionnaire. Participants were instructed to contact the research team if they had any questions or encountered technical difficulties.

To ensure the authenticity of the answers, parents were asked to supervise their children without interfering with their opinions, focusing only on clarifying doubts about the questionnaire.

After data collection, all the information was anonymized.

### 2.4 Variables and instruments

#### 2.4.1 Anthropometry and body composition

Height was assessed with a Stadiometer fixed in the anthropometric position, without shoes, after the expiratory phase and recorded with an accuracy of 0.1 cm (Height stadiometer, Sanny ES2020, American Medical do Brazil Ltda) [21].

Body weight was measured with a mechanical anthropometric scale accurate to 0.1 kg, with participants wearing as light clothing as possible, without shoes, with arms at their sides (Welmy scale, Brazil) [21].

BMI was calculated as body weight in kilograms divided by the square of height in meters [BMI = weight (kg)/height (m)^2^]. BMI z-score was calculated according to the World Health Organization reference [19] using the AnthroPlus calculator (version 1.0.4, OMS).

Body fat mass (BFM) and skeletal muscle mass (SMM) were estimated by single-frequency bioelectrical impedance analysis (Quantum II, RJL Systems, USA). Assessments were conducted with participants in the supine position, barefoot and wearing light clothing. After cleaning the skin with alcohol, four electrodes were placed on the right wrist and ankle, following standard positioning guidelines [22].

#### 2.4.2 Motives for physical activity

Motivational factors were assessed using the Portuguese version of the MPAM-R [23], adapted and validated for adolescents. The questionnaire includes 30 items distributed across five dimensions: intrinsic motivations (enjoyment and competence) and extrinsic motivations (fitness, appearance, and social) [14]. Specifically, the scale comprises seven items for enjoyment, four for competence, six for appearance, five for social interaction, and eight for fitness.

Enjoyment refers to being physically active simply because it is interesting, fun, enjoyable, and brings happiness. Competence involves acquiring new skills, tackling challenges, and improving in a particular activity. Fitness pertains to being physically active, energetic, and strong. Appearance relates to becoming more physically attractive, looking better, and achieving or maintaining a desired weight. Social interaction includes spending time with friends and meeting new people during sporting activities. Responses were rated on a 7-point Likert scale, ranging from 1 (“not true for me”) to 7 (“very true for me”). Data were collected online via Google Forms between September 2021 and March 2023. Participants reported no difficulties in understanding the instrument.

### 2.5 Statistical analysis

The normality of data distribution was assessed using the Shapiro-Wilk test and confirmed by visual inspection of histograms. Descriptive statistics are reported as means and standard deviations (Mean ± SD) for normally distributed variables, and as medians and interquartile ranges, reported as Median (IQR), for non-normally distributed variables. Between-group differences were analyzed using independent samples t-tests or Mann-Whitney tests, respectively. The relationship between participation motives and body composition was analyzed using partial correlation analyses (Pearson's coefficient), controlling for age and sex.

A quantile mixed-effects regression model using motivational factors as independent variables and body composition as dependent variables, controlling for age and sex, was additionally computed.

Data were analyzed using IBM SPSS (version 28.0, IBM, New York, USA) and R version 4.3.1. A p-value ≤ 0.05 was considered statistically significant.

## 3 Results

A total of 50 adolescents (60.0 % boys) participated in this study, with a median age of 13.6 years (IQR: 2.4) and a median BMI z-score of 3.42 (IQR: 1.55). Independent sample t-tests revealed that girls had a higher BFM (Mean Difference, MD = 4.4 %, p = 0.016) and lower SMM (MD = −4.4 ± 1.7, p = 0.016), compared to boys. No other significant differences were found between girls and boys (Table 1).

**Table 1:** Participants' baseline characteristics.

*Variable*	Total (*n* = 50)	Girls (*n* = 20)	Boys (*n* = 30)	*p-value*
Age (years)	13.6 (2.4)	13.8 (3.0)	13.3 (2.0)	.357 ^b^
Weight (kg)	103.8 (39.0)	99.1 (45.3)	105.3 (35.6)	.362 ^b^
Height (cm)	165.8 ± 9.1	162.1 ± 8.3	168.3 ± 8.9	.362^a^
BMI (kg/m^2^)	36.5 (10.8)	36.6 (11.2)	36.5 (10.7)	.866 ^b^
BMI z-score	3.42 (1.55)	3.42 (1.43)	3.54 (1.90)	.469 ^b^
BFM (%)	37.0 ± 6.4	39.6 ± 6.1	35.2 ± 6.0	0.016^a^
SMM (%)	63.0 ± 6.4	60.4 ± 6.1	64.8 ± 6.0	0.016^a^

Data is presented as mean ± SD: standard deviation; Median (interquartile range).

BFM: body fat mass; BMI: body mass index; SMM: skeletal muscle mass.

^a^^b^ Between-group differences were analyzed using independent samples t-tests (a) for normally distributed variables and Mann-Whitney U tests (b) for non-normally distributed variables.

### 3.1 Motives for engaging in the physical exercise program

Overall, the mean scores indicated that fitness was the primary motive for engaging in a PA program (6.41 ± 0.81). Enjoyment (4.98 ± 1.47), competence (4.55 ± 1.99), and appearance (5.64 ± 1.43) were identified as secondary motives. Social was the least relevant motive, presenting the lowest mean score (3.79 ± 1.85). No significant gender differences were observed (Table 2).

**Table 2:** Differences in motivation depending on sex.

Motives	Total			Girls	Boys	
	Mean ± SD	Min	Max	Mean ± SD	Mean ± SD	*p-value*
Enjoyment	4.98 ± 1.47	1.18	7.00	4.67 ± 1.66	5.19 ± 1.32	.181
Competence	4.55 ± 1.99	1.00	7.00	3.96 ± 2.16	4.94 ± 1.80	.156
Appearance	5.64 ± 1.43	1.73	7.00	5.98 ± 1.03	5.41 ± 1.61	.376
Fitness	6.41 ± 0.81	3.67	7.00	6.44 ± 0.68	6.25 ± 0.87	.057
Social	3.79 ± 1.85	1.00	7.00	3.94 ± 1.92	3.68 ± 1.82	.641

Mean ± SD: standard deviation; MPAM-R: Motives for Physical Activity Measure–Revised.

Detailed descriptive statistics for each MPAM-R item are provided in Supplemental Table 1.

### 3.2 Associations between motivational dimensions and body composition

Partial correlation analyses, controlling for age and sex, revealed negative associations of enjoyment motives (*r*_*p*_ = - 0.29; p = 0.045) and competence (*r*_*p*_ = - 0.32; p = 0.027) with body weight, indicating small to moderate effect sizes. No other motivational dimensions showed significant associations with weight (Table 3).

**Table 3:** Partial correlations between motives for physical activity and body composition in adolescents with obesity.

	Weight (kg)		BMI (kg/m2)		BMI z-score		BFM (%)	
Motives	*r*	*p-value*	*r*	*p-value*	*r*	*p-value*	*r*	*p-value*
Enjoyment	−0.29	0**.045**	−0.14	.333	−0.12	.419	−0.05	.727
Competence	−0.32	0**.027**	−0.22	.138	−0.19	.191	−0.17	.236
Appearance	0.02	.895	0.06	.672	0.07	.654	−0.02	.905
Fitness	0.01	.940	0.14	.327	0.20	.181	0.14	.347
Social	−0.14	.354	−0.07	.622	−0.05	.712	−0.04	.783

Partial correlation coefficients (r_p_) calculated using Pearson's test, controlling for age and sex.

p values < .05 were considered statistically significant and are shown in bold.

For BMI, BMI z-score, and BFM, no statistically significant associations were observed. However, enjoyment and competence tended to be negatively associated with these indicators, while fitness and appearance showed weak positive trends.

### 3.3 Analysis of motivational dimensions as predictors of body composition

Table 4 presents the regression analysis results aimed at identifying significant motivational predictors of body composition. Fitness was identified as a significant predictor for BMI z-score (β = 0.4, 95 % CI: 0.1 to 0.8, p = 0.027).Other motivational dimensions did not show significant associations with body composition indicators, although some trends mirrored the correlation analysis. Specifically, enjoyment and competence showed negative but non-significant associations with weight and BMI, while appearance, fitness, and social motives tended to show positive coefficients.

**Table 4:** Analysis of motivational dimensions as predictors of body composition.

	Weight (kg)		BMI (kg/m2)		BMI z-score		BFM (%)	
Motives	β (95 % CI)	*p-value*	β (95 % CI)	*p-value*	β (95 % CI)	*p-value*	β (95 % CI)	*p-value*
Enjoyment	−5.2 (-13.8, 3.3)	.223	−0.4 (-3.3, 2.5)	.793	−0.1 (-0.5, 0.3)	.732	0.3 (-2.6, 3.2)	.830
Competence	−4.7 (-10.1, 0.6)	.081	−1.2 (-2.9, 0.5)	.169	−0.2 (-0.4, 0.1)	.158	−1.3 (-3.4, 0.8)	.213
Appearance	2.7 (-2.7, 8.0)	.319	0.4 (-1.3, 2.2)	.634	0.1 (-0.2, 0.4)	.501	0.1 (-1.5, 1.7)	.879
Fitness	5.6 (-4.7, 16.0)	.276	2.3 (-1.0, 5.6)	.169	0.4 (0.1, 0.8)	0**.027**	1.8 (-1.4, 4.7)	.222
Social	1.0 (-4.9, 6.9)	.736	0.2 (-2.1, 2.4)	.868	−0.02 (-0.3, 0.3)	.908	0.1 (0.1, 1.7)	.850

BFM: body fat mass; BMI: body mass index.

Quantile regression for mixed models; β in bold indicates a significant interaction (p ≤ .05); β: the expected value of the median; βs standardized and adjusted for age and sex.

## 4 Discussion

Understanding the dimensions of motivation that influence PA engagement among adolescents with obesity is essential for developing effective interventions that address barriers and encourage active lifestyles [24]. In this context, the present study aimed to identify the motivational factors associated with engagement in a PA program and examine the relationship between motivation and body composition in adolescents with obesity. Study results revealed complex relationships: (1) extrinsic motivation, especially fitness-related reasons, served as the main driver of initial involvement; (2) intrinsic motivations, such as enjoyment and perceived competence, showed a negative correlation with body weight; and, (3) fitness, despite being extrinsic, was the only dimension that significantly predicted BMI z-score.

While systematic reviews highlight intrinsic motivation, especially perceived competence and personal improvement, as primary drivers of PA in adolescents [25], our findings suggest that, in adolescents undergoing clinical obesity treatment, extrinsic motives related to fitness may initially be more influential. Adolescents are often exposed to external messages emphasizing health and performance, which could explain the prominence of these motives early on in engagement [12].

Social motivation, in turn, showed the lowest relevance for PA engagement. This aligns with previous evidence suggesting that adolescents with obesity often avoid group environments due to fear of judgment or negative social experiences [18]. In clinical settings, where stigma and social comparison may be intensified, the social environment can serve as a barrier rather than a facilitator. However, other studies indicate that inclusive and supportive environments may reverse this trend, encouraging engagement through peer support [26]. These differences emphasize the importance of creating psychologically safe spaces and promoting cooperative activities to turn social motivation into a positive driving force for engagement in this population [27].

Although no statistically significant sex differences were found, exploratory trends aligned with previous studies suggesting potential variations in motivational profiles between boys and girls [28,29]. These observations, while preliminary, underscore the value of considering individual sex-sensitive motivational patterns when designing PA interventions for adolescents with obesity.

The negative associations of enjoyment and competence with body weight highlight the potential role of intrinsic motivation in weight management. These findings are consistent with research showing that enjoyment of PA and perceived competence promote higher activity levels and healthier behaviors [16,30]. The satisfaction of psychological needs through positive experiences has been consistently linked to increased motivation and sustained engagement [31,32]. The SDT supports this, proposing that while extrinsic reasons may initiate behavior, the long-term maintenance of 10.13039/100006131PA depends on developing intrinsic motivation [33]. Thus, interventions should combine both elements: leveraging extrinsic motives, such as improving fitness, and progressively fostering intrinsic motivation to promote sustainable engagement [34].

As noted earlier, fitness dimension was the only significant predictor of BMI z-score, suggesting that adolescents driven by physical conditioning goals may achieve more favorable body composition indicators. This likely reflects the direct link between fitness-oriented goals and health outcomes. Therefore, reinforcing fitness-related goals while nurturing intrinsic motives may be an effective strategy to support sustainable 10.13039/100006131PA engagement, weight management, and improved well-being in adolescents with obesity [35].

## 5 Limitations

This study has limitations that should be considered when interpreting the results. For instance, participants were recruited from a specialized outpatient obesity clinic, where the PA program was implemented as part of a multidisciplinary clinical approach. This setting may have shaped the reported motivational factors, emphasizing extrinsic motives such as physical fitness and appearance. Therefore, the results may not be generalizable to adolescents with obesity who engage in PA in school, community, or self-initiated settings.

While the MPAM-R is a validated instrument, the use of self-report questionnaires may introduce report bias, such as social desirability or contextual influence. Nevertheless, prior research indicates that, when administered under standard conditions and ensuring participant anonymity, self-reports can reliably capture motivational constructs in health-related contexts [27]. In this study, we implemented the identified procedures to minimize potential bias and enhance data reliability.

An important limitation is the relatively small sample size, which reduces statistical power to detect smaller effects and may affect the generalizability of our findings. *A priori* power analysis was not performed, as the study used secondary data from a trial not originally designed to test these specific associations. Given the cross-sectional design and exploratory nature of the analyses, the sample size was constrained by the available eligible participants. Nevertheless, although no *a priori* power analysis was performed, similar sample sizes have been used in comparable clinical studies involving adolescents with obesity [36,37]. Future research should confirm these findings in larger, more diverse groups to improve external validity and enable more advanced analyses of potential moderators.

Another possible limitation may be the absence of other covariates known to influence motivational profiles and body composition, such as psychological factors (e.g., self-efficacy, body image), socioeconomic context, and previous PA experiences.

Although these limitations require caution when interpreting the results, it brings novelty to adolescent obesity treatment, also highlighting the need for more studies on the topic.

## 6 Conclusion

• Extrinsic motives like fitness and appearance drive PA engagement in treatment-seeking adolescents with obesity.
• Intrinsic motives, especially enjoyment and competence, were inversely associated with BMI z-score in this sample.
• Targeting both fitness goals and intrinsic motivation may help improve adherence and weight outcomes in obesity care.

More studies with larger samples and longitudinal or interventional designs are needed to confirm these findings.

## Author contributions

Conceptualization, FF, and AVS.; data curation, FF, and AVS.; formal analysis, FF.; funding acquisition, MARGM, and MAB; investigation, MRZ, and FF; methodology, FF, AVS and MAB; project administration, MAB; resources, MAB; supervision, AVS and MAB.; validation, AVS and MAB; visualization, MRZ, MARGM and MAB.; writing—original draft, FF.; writing—review and editing, FF, AVS, MRZ, MARGM, and MAB. All authors have read and agreed to the published version of the manuscript.

## Ethics

This study was approved by the Research Ethics Committee of the State University of Campinas under opinion number 4.940.045, following the norms established by Resolution 466/2012 of the National Health Council. All participants and their guardians who agreed to participate signed a free and informed consent form.

## Artificial intelligence (AI)

During the preparation of this work the authors did not use AI-assisted technologies.

## Funding

This work did not receive any specific grant from funding agencies in the public, commercial, or not-for-profit sectors. However, Fábio de Freitas received a scholarship from the Coordination for the Improvement of Higher Education Personnel (CAPES), Brazil (Finance Code 001), which supported his academic training during the development of this research.

## Declaration of competing interest

The authors declare that they have no known competing financial interests or personal relationships that could have appeared to influence the work reported in this paper.
