# Supplementary material for: Cross-sectional associations between intrinsic and extrinsic motivation for physical activity and body composition in adolescents with obesity
Source: Obes Pillars. 2025 Jul 19;16:100197. doi: 10.1016/j.obpill.2025.100197 (PMC12310392; doi:10.1016/j.obpill.2025.100197)
Supplement: Multimedia component 1 [file mmc1.docx]

**Supplemental Table 1.** Evaluation of the specific motives for the Motives for Physical Activity Measure-Revised scale.

| **Motives** | **Mean** | **± SD** | **Minimum** | **Maximum** |
| --- | --- | --- | --- | --- |
| **Enjoyment** |  |  |  |  |
| 2. Because it is pleasurable. | 4.96 | 1.76 | 1 | 7 |
| 7. Because I like to practice this activity. | 4.76 | 1.92 | 1 | 7 |
| 8. Because I want to improve the skills I already have. | 4.90 | 1.97 | 1 | 7 |
| 11. Because I'm happy. | 5.48 | 1.49 | 2 | 7 |
| 12. Because I want to maintain my current skill level. | 4.42 | 2.24 | 1 | 7 |
| 18. Because I think it's interesting. | 5.36 | 1.72 | 1 | 7 |
| 22. Because I like this activity. | 4.98 | 1.83 | 1 | 7 |
| 26. Because I find this activity stimulating. | 5.02 | 1.80 | 1 | 7 |
| 29. Because I enjoy the pleasure of participating. | 4.74 | 2.01 | 1 | 7 |
|  |  |  |  |  |
| **Competence** |  |  |  |  |
| 3. Because I like to practice physically challenging activities. | 4.24 | 2.19 | 1 | 7 |
| 4. Because I want to learn new skills. | 5.08 | 1.91 | 1 | 7 |
| 9. Because I like the challenge. | 4.78 | 2.28 | 1 | 7 |
| 14. Because I enjoy physically challenging activities. | 4.34 | 2.26 | 1 | 7 |
|  |  |  |  |  |
| **Appearance** |  |  |  |  |
| 5. Because I want to lose or maintain weight and look better. | 6.66 | 0.89 | 3 | 7 |
| 10. Because I want to define my muscles and look better. | 5.66 | 2.06 | 1 | 7 |
| 17. Because I want to improve my appearance. | 6.20 | 1.54 | 1 | 7 |
| 20. Because I want others to find me attractive. | 4.58 | 2.38 | 1 | 7 |
| 24. Because I want to improve my body shape. | 6.40 | 1.29 | 1 | 7 |
| 25. Because I want to improve in my activity. | 5.68 | 1.63 | 1 | 7 |
| 27. Because I think I'm physically ugly if I don't. | 4.60 | 2.36 | 1 | 7 |
|  |  |  |  |  |
| **Fitness** |  |  |  |  |
| 1. Because I want to get physically fit. | 6.20 | 1.41 | 2 | 7 |
| 13. Because I want to have more energy. | 6.44 | 1.07 | 2 | 7 |
| 16. Because I want to improve my cardiovascular condition. | 6.18 | 1.38 | 1 | 7 |
| 19. Because I want to maintain my physical strength to lead a healthy life. | 6.44 | 1.23 | 1 | 7 |
| 23. Because I want to maintain my physical health and well-being. | 6.58 | 0.76 | 4 | 7 |
|  |  |  |  |  |
| **Social** |  |  |  |  |
| 6. Because I want to meet my friends. | 3.62 | 2.30 | 1 | 7 |
| 15. Because I enjoy the company of others who are interested in this activity. | 4.18 | 2.15 | 1 | 7 |
| 21. Because I want to meet new people. | 4.14 | 2.29 | 1 | 7 |
| 28. Because my friends want me to do it. | 2.78 | 2.26 | 1 | 7 |
| 30. Because I enjoy spending time with other people practicing this activity. | 3.94 | 2.22 | 1 | 7 |

Mean ± SD: standard deviation; MPAM-R: Motives for Physical Activity Measure-Revised
